# Supplementary material for: Efficient alkane oxidation under combustion engine and atmospheric conditions
Source: Commun Chem. 2021 Feb 18;4:18. doi: 10.1038/s42004-020-00445-3 (PMC9814728; doi:10.1038/s42004-020-00445-3)
Supplement: Supplementary file 1 — Supplementary Information [file 42004_2020_445_MOESM1_ESM.pdf]

Supplementary Information for

Efficient alkane oxidation under combustion engine and atmospheric conditions

Zhandong Wang[1,2,3#]*, Mikael Ehn[4#], Matti P. Rissanen[4, 5], Olga Garmash[4], Lauriane Quéléver[4], Lili Xing[6], Manuel Monge-Palacios[3], Pekka Rantala[4], Neil M. Donahue[7], Torsten Berndt[8], S. Mani Sarathy[3]*

[1] National Synchrotron Radiation Laboratory, University of Science and Technology of China, Hefei, Anhui 230029, P. R. China

[2] State Key Laboratory of Fire Science, University of Science and Technology of China, Hefei, Anhui 230026, PR China

[3] King Abdullah University of Science and Technology (KAUST), Clean Combustion Research Center (CCRC), Thuwal 23955-6900, Saudi Arabia

[4] Institute for Atmospheric and Earth System Research (INAR), University of Helsinki, Helsinki 00014, Finland

[5] Aerosol Physics Laboratory, Physics Unit, Faculty of Engineering and Natural Sciences, Tampere University, 33720 Tampere, Finland

[6] Energy and Power Engineering Institute, Henan University of Science and Technology, Luoyang, Henan 471003, China

[7] Center for Atmospheric Particle Studies, and Department of Chemistry, Department of Chemical Engineering, Department of Engineering and Public Policy, Carnegie Mellon University, Pittsburgh, PA 15213, USA

[8] Leibniz Institute for Tropospheric Research (TROPOS), Atmospheric Chemistry Dept. (ACD), 04318 Leipzig, Germany

*Correspondence to: Zhandong Wang (zhdwang@ustc.edu.cn); Mani Sarathy (mani.sarathy@kaust.edu.sa)

[#] These authors contributed equally

Table of content

Supplementary Note 1: Peroxy radical chemistry and HOM

Supplementary Note 2: Alkoxy radical chemistry

Supplementary Note 3: Combustion autoxidation

Supplementary Note 4: Transition from combustion autoxidation to atmospheric autoxidation

Supplementary Note 5: Atmospheric autoxidation initiated by TME+O_3

Supplementary Note 6: Estimation of HOM yield in Helsinki flow reactor data

Supplementary Note 7: Kinetic modelling of decanal and decalin oxidation in flow reactors

Supplementary Note 8: Autoxidation of decalin, cyclohexane, and *n*-decane in the presence of NO conducted in the Leipzig flow reactor

Supplementary References

Supplementary Note 1: Peroxy radical chemistry and HOM

The autoxidation process is governed by the formation of peroxy radicals (RO_2), which undergo multiple steps of H-shift and O_2 addition to form more highly oxygenated molecules. These sequential oxidation steps are favorable when the peroxy radicals contain certain functional groups such as carbonyl, alcohol, and tertiary C-Hs, or when the reaction temperature is high enough to favor the H-shift reactions [1,2]. The general scheme of the autoxidation process is shown in Supplementary Scheme 1. This process has been observed during the atmospheric autoxidation of biogenic VOC initiated by OH and O_3 [3,4] and anthropogenic VOC such as aromatics [5], as well as in combustion autoxidation of hydrocarbons and oxygenated fuels [6].

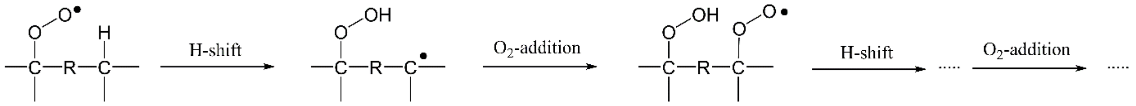

Supplementary Scheme 1. The general reaction scheme of peroxy radical autoxidation chemistry.

Under atmospheric conditions, oxidation of alkanes is initiated via H-atom abstraction by OH radicals, forming RO_2 radicals after O_2 addition. Past research has shown that bimolecular reactions with HO_2, NO, or other RO_2, are dominant reaction pathways for the peroxy radicals [7]. The general scheme for these bimolecular reactions is shown below, which has radicals in black and closed shell species in gray. The rate constant for these reactions is given using the peroxy radical from *n*-hexane as an example [8]. Corresponding reactions and rate constants for a substituted RO_2 radical are given in Scheme 2. The rate constant for the formation of ROOR accretion products is not known, and will likely depend strongly on the reaction partner structure [9].

$$\boldsymbol{RO_2 + NO \rightarrow RO + NO_2} \quad (8\times10^{-12}\ \mathrm{cm^3/molecule/s})$$
$$\rightarrow \boldsymbol{RONO_2} \quad (7\times10^{-13}\ \mathrm{cm^3/molecule/s})$$
$$\boldsymbol{RO_2 + HO_2 \rightarrow ROOH + O_2}\ (1.8\times10^{-11}\ \mathrm{cm^3/molecule/s})$$

$$\boldsymbol{RO_2 + RO_2 \rightarrow RO + RO + O_2}\ (7.8\times10^{-13}\ \mathrm{cm^3/molecule/s})$$
$$\rightarrow \boldsymbol{ROH + R(O) + O_2}\ (2.6\times10^{-13}\ \mathrm{cm^3/molecule/s})$$
$$\rightarrow \boldsymbol{ROOR}\ \mathbf{(?)}$$

In a recent review, Bianchi et al. [10] recommended that the term HOM, standing for highly oxygenated organic molecules, be used to denote a specific subset of atmospheric oxidation products. By their definition, HOM are molecules formed by autoxidation involving RO_2, under atmospherically relevant conditions. In addition, they should typically contain 6 or more O-atoms. Therefore, many of the important products identified in this work cannot be classified as HOM, since they were either formed at elevated temperatures and/or the final products included less than 6 O-atoms.

Supplementary Note 2: Alkoxy radical chemistry

The alkoxy radical (RO) is an important intermediate during alkane oxidation [11-13]. In the following section, the reaction pathways of RO radicals from different precursor VOC structures are discussed.

(1) Linear and branched alkanes

As an example, we discuss the possible fates of the RO radical where the alkoxy group is located on the terminal carbon in the *n*-hexyl chain. The possible reactions of this RO radical are shown in Supplementary Scheme 2. The rate constants for important reactions are from the MCM [8] and/or literature review [14]. The unit is s^{-1} for unimolecular reactions and cm^3/molecule/s for bimolecular reactions. The radicals are in black and closed shell species are in gray. This RO radical can i) undergo β-C-C scission to form an aldehyde and an alkyl radical, ii) react with O_2 to form a carbonyl and HO_2, or iii) undergo an H-shift followed by O_2 addition to form a RO_2 with an –OH substitution (OH-ROO). The OH-ROO radical can then undergo bimolecular reactions with HO_2, NO and RO_2, leading to the closed shell products and a new RO radical with an –OH substitution (OH-RO). The dominant reaction for this RO radical is isomerization followed by loss of HO_2 to form the closed shell product OH-R=O.

In the case of a branched alkane, the reaction mechanisms are similar, with the exception that the branching can facilitate H-abstractions from the tertiary C, as well as increasing the likelihood of the scission reaction of the RO.

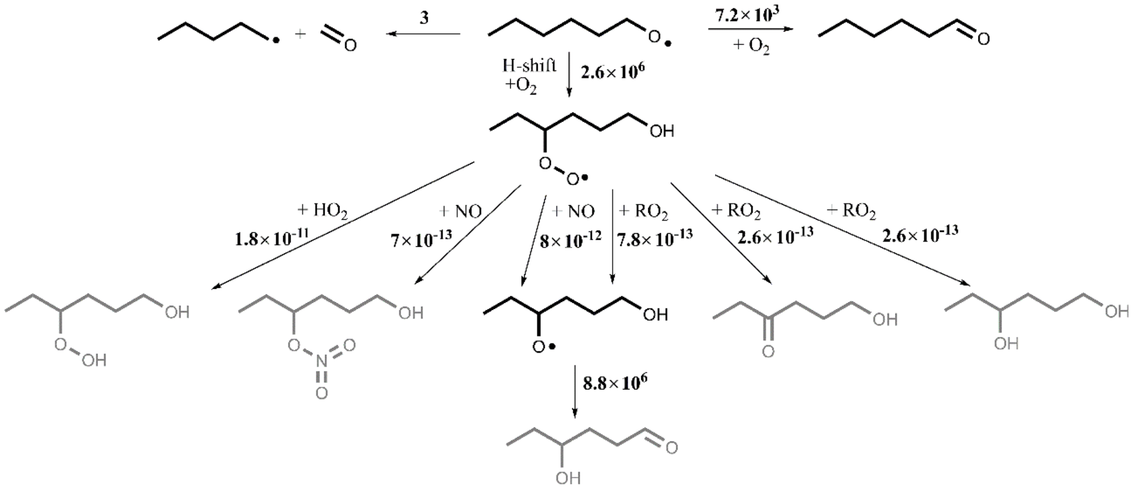

Supplementary Scheme 2. The reaction scheme of an RO radical from *n*-hexane. The unit is s^{-1} for unimolecular reactions and cm^3/molecule/s for bimolecular reactions. The radicals are in black and closed shell species in gray. The rate constant of alkoxy radicals with O_2 was estimated using an O_2 concentration of 0.2 atm and a value of 1.4×10^{-14} cm^3/molecule/s for the O_2 reaction with the alkoxy radical (refers to *n*-propoxy [14]).

(2) Cycloalkanes

Cyclohexane is used here to illustrate the general RO reaction mechanisms for cycloalkanes (Supplementary Scheme 3), with rate constants from the MCM [8]. The radicals are in black and closed shell species in gray. This RO radical can react with O_2 to form a carbonyl, but the dominant pathway is the β-C-C scission to form a carbon-centered radical with an aldehyde functionality, followed by O_2 addition to form an RO_2. This O=C-ROO radical can then undergo

bimolecular reactions with HO_2, NO and RO_2, leading to closed shell products or another RO radical (O=C-RO). The RO radical can undergo an internal H-shift followed by O_2 addition, leading to a new RO_2 (O=C-ROO-OH), with similar available reaction pathways as the O=C-ROO radical.

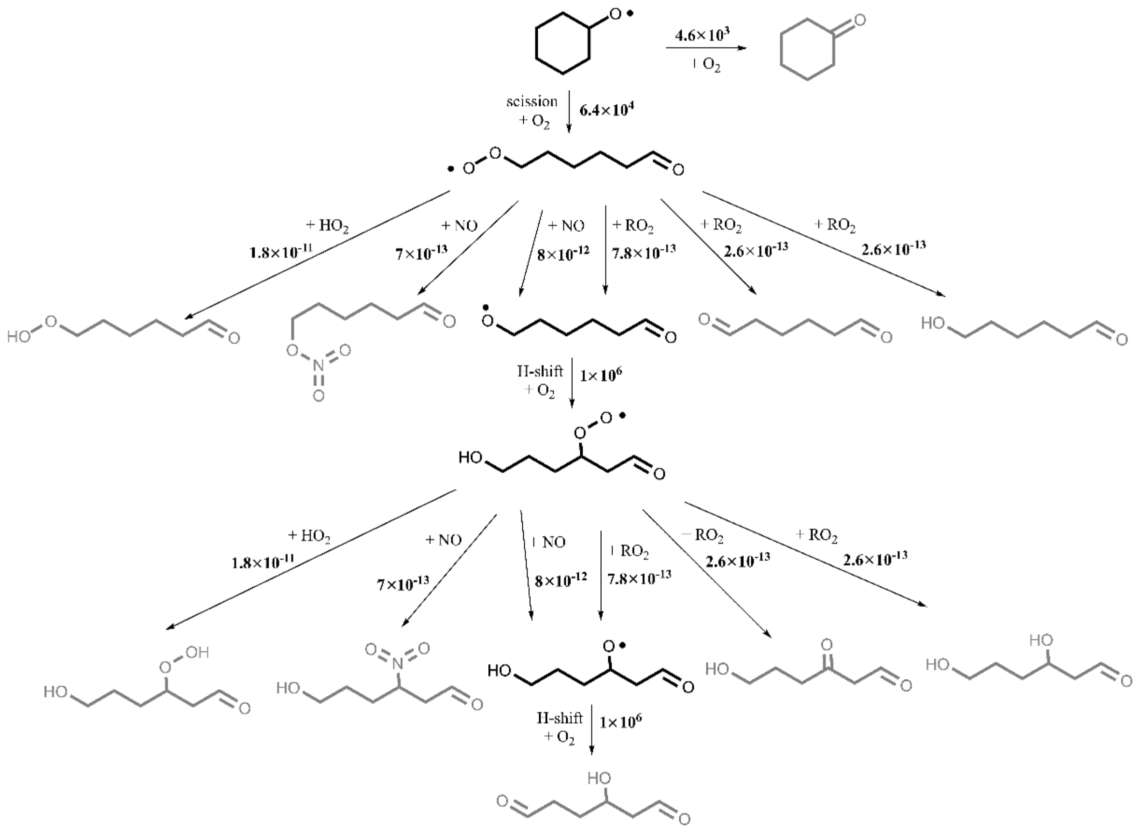

Supplementary Scheme 3. The reaction scheme of an RO radical from cyclohexane. The unit is s^{-1} for unimolecular reactions and cm^3/molecule/s for bimolecular reactions. The radicals are in black and closed shell species in gray. The rate constant of alkoxy radicals with O_2 was estimated using an O_2 concentration of 0.2 atm and a value of 9×10^{-15} cm^3/molecule/s for the O_2 reaction with the alkoxy radical.

(3) Aldehydes

In the case of aldehydes, due to their weak C-H bond in the carbonyl group, only the H-abstraction reaction from the HC=O site is considered in the MCM mechanism [8]. When peroxy radicals derived from an aldehyde undergo bimolecular reaction, the formed RO radical is an acyloxy radical. The dominant consumption pathway for acyloxy radicals is dissociation into CO_2 and an alkyl radical (Supplementary Scheme 4). The rate constant is estimated from the decarboxylation reaction of CH_2CO_2 radical [15]. The alkyl radical then reacts with O_2 and forms a peroxy radical, whose reaction scheme is similar to the ones described above for *n*-hexane.

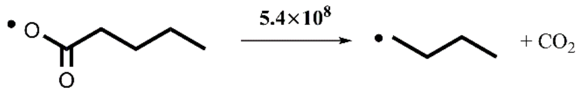

Supplementary Scheme 4. The reaction scheme of an RO radical from pentanal. The unit is s^{-1} for unimolecular reactions. The radicals are in black and closed shell species in gray.

In our work, we observed a large number of oxidation products with the same carbon number as the precursor aldehydes, indicating that the H-atom abstraction by OH from other carbon sites is also possible. A more detailed discussion on aldehyde oxidation pathways are given in Supplementary Note 5.

(4) Alcohol

In the case of alcohols, H-atom abstraction of the C-H adjacent (alpha) to the –OH group is favored due to the low bond dissociation energy. The alpha-hydroxyalkyl radical can react with O_2 to form an aldehyde and HO_2. An H-atom abstraction from any other position along the carbon chain will form RO_2 radicals. The formed RO_2 can again undergo bimolecular reactions with HO_2, NO and RO_2. These reactions produce closed shell products or a RO radical with an –OH substitution (OH-RO). The reactions of OH-RO from *n*-butanol oxidation is shown in Supplementary Scheme 5. The rate constants are from the MCM [8]. The OH-RO radical can undergo dissociation to an aldehyde and an alcohol radical, or react with O_2 to form a hydroxycarbonyl compound.

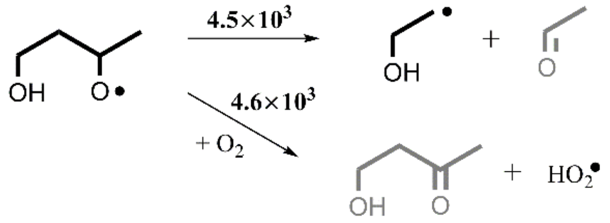

Supplementary Scheme 5. The reaction scheme of an RO radical from *n*-butanol. The unit is s^{-1} for unimolecular reactions and cm^3/molecule/s for bimolecular reactions. The radicals are in black and closed shell species in gray. The rate constant of alkoxy radicals with O_2 was estimated using an O_2 concentration of 0.2 atm and a value of 9×10^{-15} cm^3/molecule/s for the O_2 reaction with the alkoxy radical.

Supplementary Note 3: Combustion autoxidation

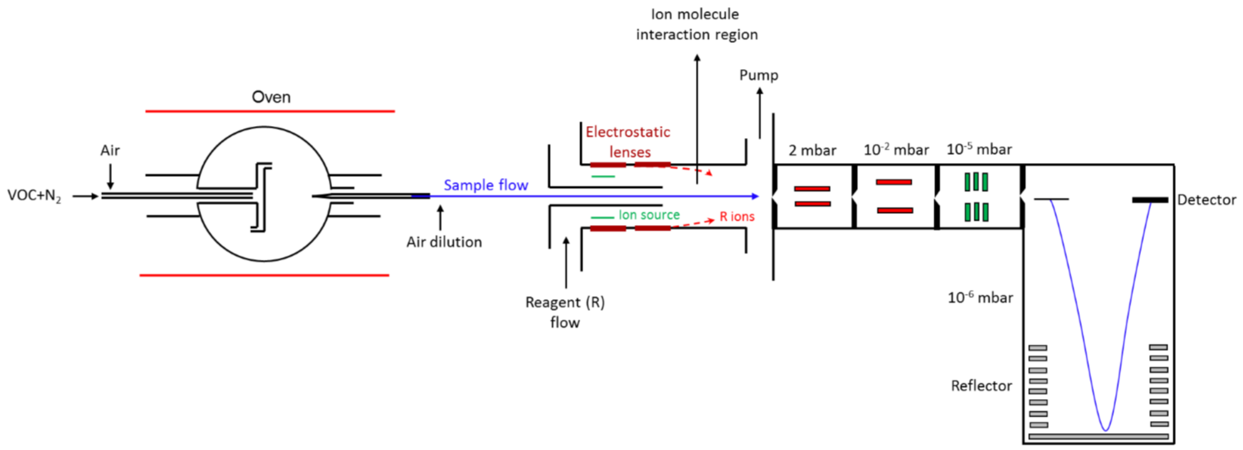

Supplementary Figure 1. Schematic of the system to study VOC combustion autoxidation.

In combustion autoxidation, the first step of the reaction is the bimolecular reaction of hydrocarbon with O_2, i.e., O_2 is able to abstract H-atoms and initiates the autoxidation of decanal at 520 K. Although this initiation reaction is very slow, it often produces an OH radical in the process, which can initiate further oxidation of hydrocarbons. Thus, the oxidation of the fuel/VOC is driven by both O_2 and OH; the contribution of OH initiation is much higher than the O_2 initiation [16,17]. The mass spectra measured during the autoxidation of *n*-decane, 2,7-dimethyloctane, *n*-butylcyclohexane, and 2-decanone are shown in Supplementary Figure 2. The mass peaks corresponding to the third and fourth O_2 addition reactions were detected.

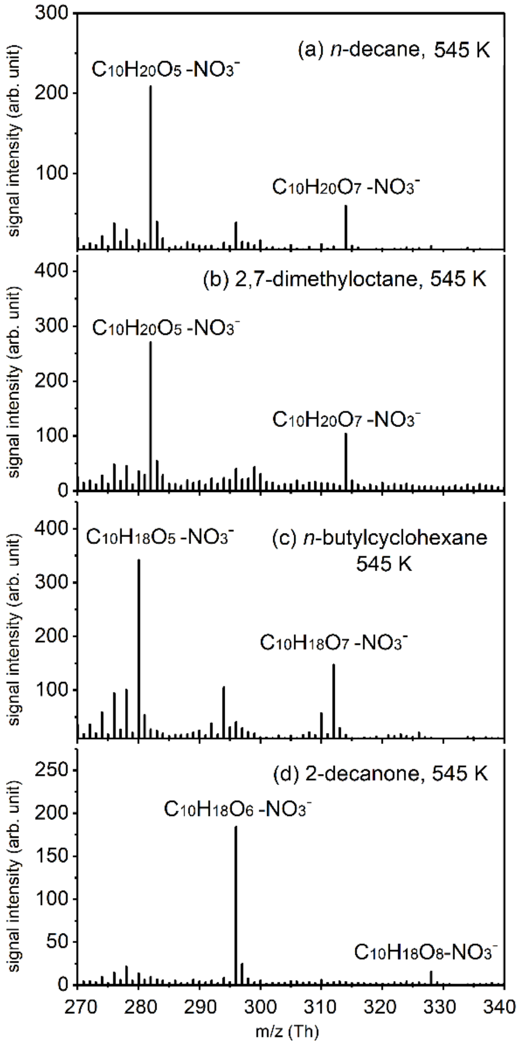

Supplementary Figure 2. Mass spectra measured during the autoxidation of *n*-decane (a), 2,7-dimethyloctane (b), *n*-butylcyclohexane (c), and 2-decanone (d).

Specifically, Supplementary Figure 3a presents the mass peaks in the range of 220 and 310 from decanal autoxidation investigated by SVUV-PIMS [6]. Apart from the mass peak corresponding to $C_{10}H_{18}O_6$, no other peak from further O_2 addition was detected. The mass peak at 278 Th is a background signal. In the autoxidation experiment of decanal by CI-APi-TOF, the mass peak of $C_{10}H_{18}O_6$ was also observed. This further confirmed previous observations of third O_2

addition reaction pathways. In addition, the mass peaks of $C_{10}H_{18}O_8$ and $C_{10}H_{18}O_{10}$ were observed, as shown in Supplementary Figure 3b. The detection of these two oxygenated intermediates indicates the fourth and fifth O_2 addition reactions.

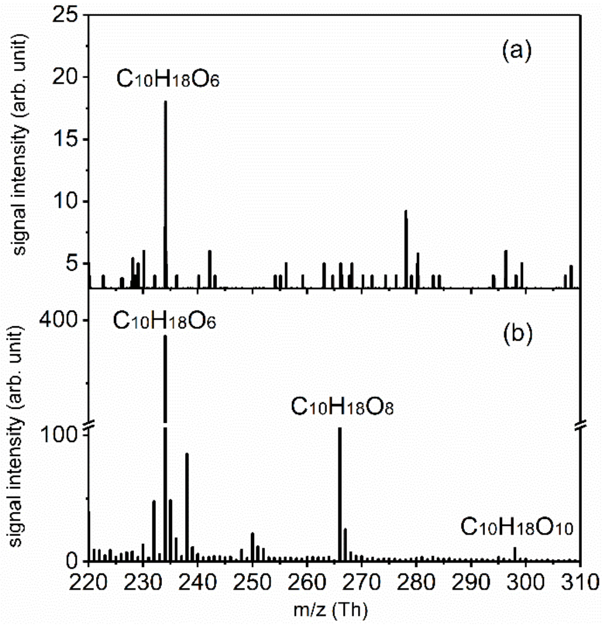

Supplementary Figure 3. Mass spectra measured during the autoxidation of decanal at 520 K. (a) SVUV-PIMS result [6] , (b) CI-APi-TOF result, the mass in (b) was subtracted by 62 (the mass of NO_3^-) for a better comparison.

Supplementary Note 4: Transition from combustion autoxidation to atmospheric autoxidation

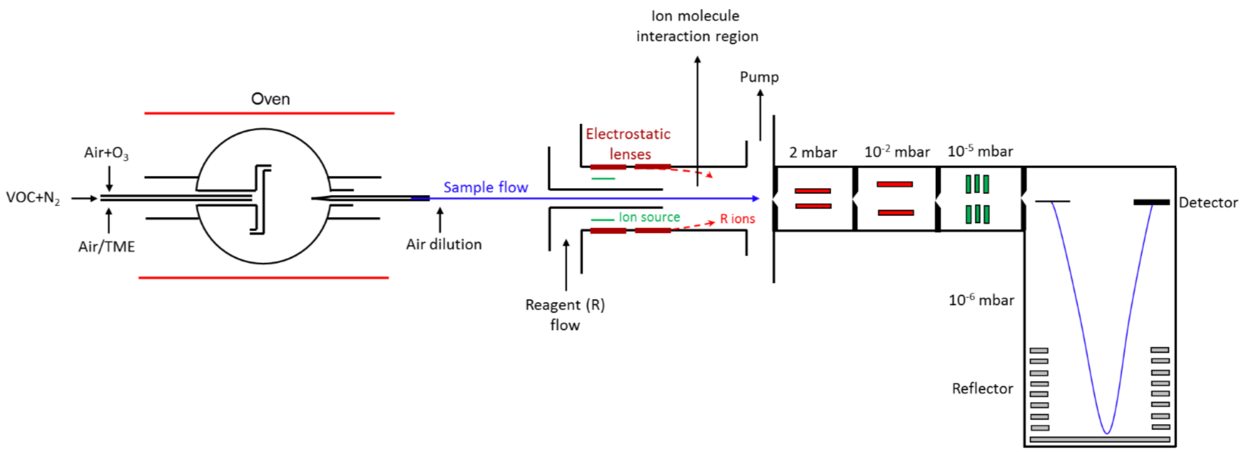

Supplementary Figure 4. Schematic of the system to study the transition from combustion autoxidation to atmospheric autoxidation. The OH radical was generated by the reaction of TME + O_3.

The observation of $C_{10}H_{18}O_6$, $C_{10}H_{18}O_8$ and $C_{10}H_{18}O_{10}$ in this work, combined with measurements of $C_{10}H_{18}O_2$, $C_{10}H_{18}O_4$, and $C_{10}H_{18}O_6$ by SVUV-PIMS in previous work [6], shows that the autoxidation of decanal under combustion related conditions involves up to five steps of internal H-shifts followed by O_2 additions. The relevant peroxy radicals in this scheme are $C_{10}H_{19}O_{3,5,7,9,11}$ (Supplementary Scheme 6).

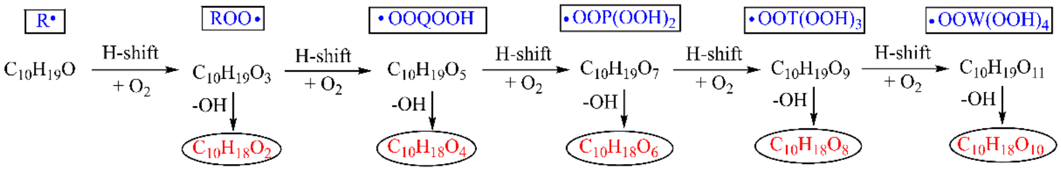

Supplementary Scheme 6. Autoxidation scheme of decanal under combustion conditions. The species labeled in blue are the generalized radical formulas, while the species labeled in red are closed shell products.

With a decrease in temperature, the results in Fig. 2 of the main text show a transition of the "pure" RO_2 H-shift chemistry towards a combination of RO_2 and RO chemistry. Supplementary Figure 5 shows decanal autoxidation data under different OH concentration in the Helsinki flow reactor at 300 K. The monomers of $C_{10}H_xO_y$ and ROOR accretion products of $C_{13}H_xO_y$ and $C_{20}H_xO_y$ are presented. The possible structure and formation mechanism for these products are discussed at the end of this section.

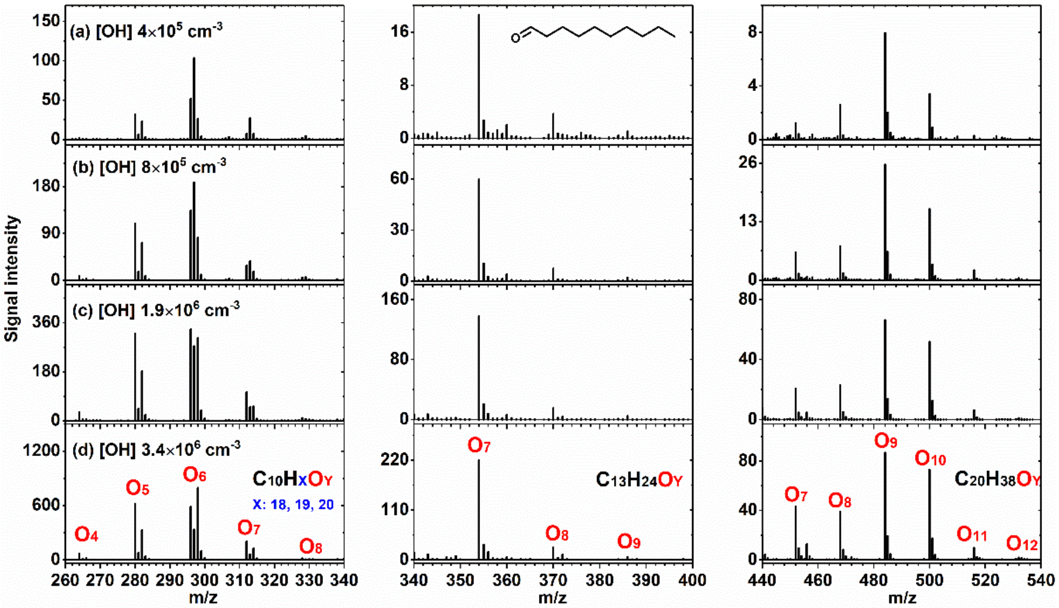

Supplementary Figure 5. Mass spectra of decanal autoxidation in the Helsinki flow reactor at 300 K under different OH concentrations, varied by adding different amounts of TME, measured by the CI-APi-TOF. The left hand panels depict monomers and the middle and right hand panels the accretion product ranges for the same experiments. The accretion products in the middle panels are produced from the bimolecular reactions of the $C_3H_5O_3$ peroxy radical from TME ozonolysis with the peroxy radicals from decanal autoxidation. The accretion products on the right are produced from the cross reactions of peroxy radicals from decanal.

As presented in the discussion above on aldehydes, H-atom abstraction of the C-H at the carbonyl group is the most

favorable pathway. However, the discussion in Supplementary Note 2 based on conventional knowledge shows that the peroxy radical formed from this radical leads to an acyloxy radical, which decomposes into CO_2 and the smaller alkyl radical. However, this conventional pathway could not explain our observations of HOM intermediates with the same carbon skeleton of decanal, such as the RO_2 radicals in Fig. 2d, $C_{10}H_{19}O_6$, $C_{10}H_{19}O_7$, and $C_{10}H_{19}O_8$. To gain more insight into a possible formation pathway, we added D_2O to the reaction system. As shown in Supplementary Figure 6, the mass peak of $C_{10}H_{19}O_6$ is primarily shifted by 2 Dalton, indicating that two H-atoms have been replaced by D-atoms. This suggests that $C_{10}H_{19}O_6$ has two labile H atoms, meaning that two H were bound to O atoms [18]. Other isomers may be present, exchanging more or less H-atoms, but due to the presence of $C_{10}H_{18}O_6$ and $C_{10}H_{20}O_6$, we cannot draw further conclusions on these. For the $C_{10}H_{19}O_7$ and $C_{10}H_{19}O_8$ radicals, the identification of a mass shift was not possible due to very weak signal intensities.

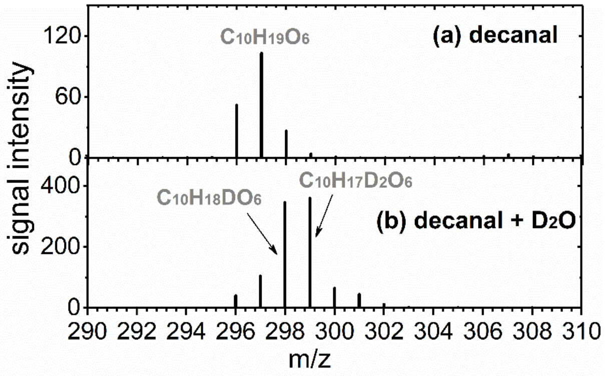

Supplementary Figure 6. Mass spectra of decanal autoxidation without (a) and with D_2O addition (b), highlighting the $C_{10}H_{19}O_6$ radical. The shift of the highest peak from 297 to 299 suggests that at least the main isomer of this radical has two labile H atoms.

Based on the information above on detected molecules, and the amount of labile H-atoms, we have some indications of how the molecules and radicals may look, and can draw up a potential reaction scheme. Firstly, we propose that the HOM formation is initiated by H-atom abstraction from one of the secondary carbon atoms. Once the first RO_2 ($C_{10}H_{19}O_3$) radical is formed, it can undergo one of two kinds of reactions: an H-shift and subsequent O_2 addition, or a bimolecular reaction with another peroxy radical. If this latter reaction forms an RO radical with a carbonyl group ($C_{10}H_{19}O_2$), it can also undergo an H-shift and O_2 addition to reform an RO_2 ($C_{10}H_{19}O_4$). This reaction pattern of reforming new radicals, via H-shifts of either RO or RO_2 radicals, can continue, leading to increasingly more oxygenated species. However, at every step, both the bimolecular reactions and the H-shifts (especially when abstracting from an already oxygenated C-atom) may lead to radical termination. This is the main challenge, to avoid all the potential termination reactions, when trying to construct an explicit mechanism by which up to 7 O-atoms can be added to the C_{10} backbone. Supplementary Scheme 7 gives some example pathways leading to the observed radical species, with a structure for $C_{10}H_{19}O_6$ that has two labile H-atoms. However, even though this scheme matches our observations, it is unlikely that exactly this pathway would be able to produce HOM with a yield of a few percent. The exact autoxidation progression thus remains largely an open question.

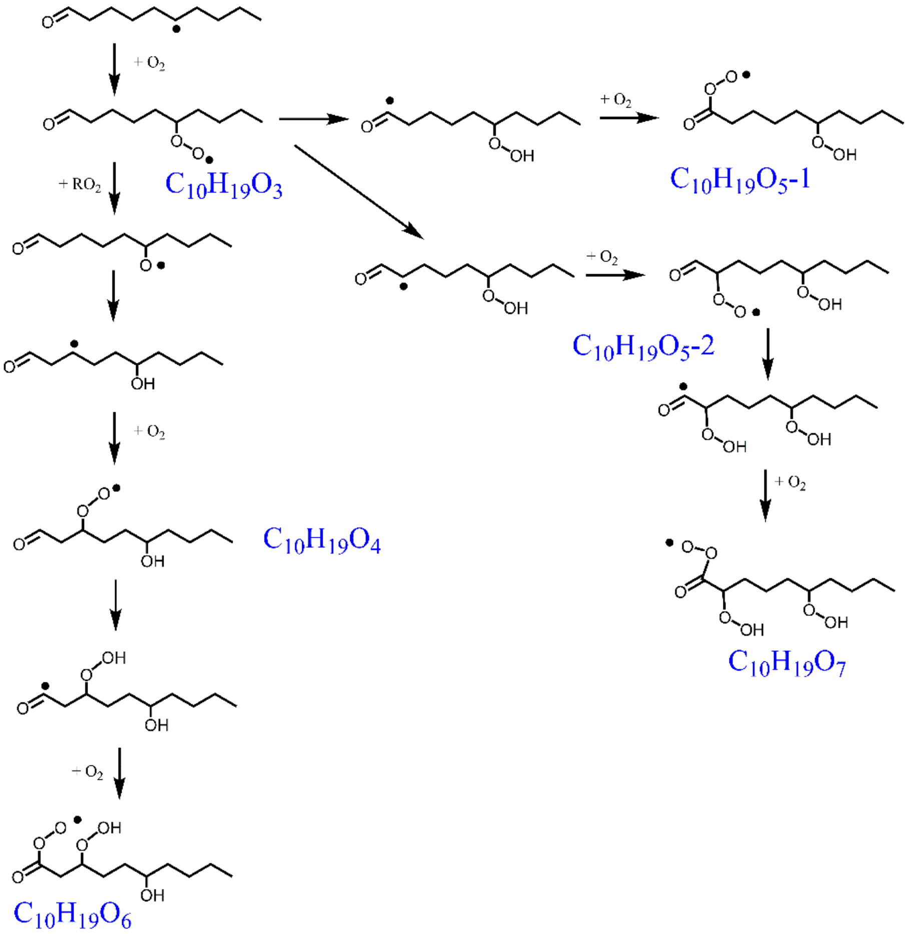

Supplementary Scheme 7. One possible reaction network of decanal autoxidation, which could form the $C_{10}H_{19}O_6$ and $C_{10}H_{19}O_7$ radicals observed in Fig. 2d. The $C_{10}H_{19}O_8$ radical may form from an additional RO_2 H-shift, or may require an additional RO isomerization step. While this scheme matches our experimental observations, we acknowledge that there are several competing pathways for almost all reaction steps, and the theoretical understanding of how to reach such high oxygen content in the decanal + OH system requires further explorations.

While the mechanistic understanding is lacking, the mass spectra are still internally consistent. For example, in Fig. 2d, the mass peak corresponding to $C_{13}H_{24}O_7$ is observed. With increasing OH concentration, another two mass peak of $C_{13}H_{24}O_8$ and $C_{13}H_{24}O_9$ became evident, as shown in Supplementary Figure 5d. These three accretion products could be produced from the bimolecular reaction of the $C_3H_5O_3$ radical ($CH_3C(O)CH_2O_2$) with the $C_{10}H_{19}O_6$, $C_{10}H_{19}O_7$, and $C_{10}H_{19}O_8$ radicals. The largest peak is from $C_{13}H_{24}O_7$, which is consistent with the largest radical $C_{10}H_{19}O_6$, in the monomer range (remembering that ROOR formation includes the concurrent formation of O_2). The observation of these ROOR accretion products is thus further evidence for the formation of $C_{10}H_{19}O_6$, $C_{10}H_{19}O_7$, and $C_{10}H_{19}O_8$ radicals.

$$\mathbf{C_{10}H_{19}O_6} + C_3H_5O_3 = C_{13}H_{24}O_7 + O_2$$
$$\mathbf{C_{10}H_{19}O_7} + C_3H_5O_3 = C_{13}H_{24}O_8 + O_2$$
$$\mathbf{C_{10}H_{19}O_8} + C_3H_5O_3 = C_{13}H_{24}O_9 + O_2$$

Furthermore, the ROOR accretion products of $C_{20}H_{38}O_7$, $C_{20}H_{38}O_8$, $C_{20}H_{38}O_9$, $C_{20}H_{38}O_{10}$, $C_{20}H_{38}O_{11}$ and $C_{20}H_{38}O_{12}$

were also observed in Supplementary Figure 5d. They could be produced from one of the following pathways:

$$\mathbf{C_{10}H_{19}O_3} + \mathbf{C_{10}H_{19}O_6} = C_{20}H_{38}O_7 + O_2$$
$$\mathbf{C_{10}H_{19}O_3} + \mathbf{C_{10}H_{19}O_7} = C_{20}H_{38}O_8 + O_2$$
$$\mathbf{C_{10}H_{19}O_5} + \mathbf{C_{10}H_{19}O_5} = C_{20}H_{38}O_8 + O_2$$
$$\mathbf{C_{10}H_{19}O_3} + \mathbf{C_{10}H_{19}O_8} = C_{20}H_{38}O_9 + O_2$$
$$\mathbf{C_{10}H_{19}O_5} + \mathbf{C_{10}H_{19}O_6} = C_{20}H_{38}O_9 + O_2$$
$$\mathbf{C_{10}H_{19}O_5} + \mathbf{C_{10}H_{19}O_7} = C_{20}H_{38}O_{10} + O_2$$
$$\mathbf{C_{10}H_{19}O_6} + \mathbf{C_{10}H_{19}O_6} = C_{20}H_{38}O_{10} + O_2$$
$$\mathbf{C_{10}H_{19}O_5} + \mathbf{C_{10}H_{19}O_8} = C_{20}H_{38}O_{11} + O_2$$
$$\mathbf{C_{10}H_{19}O_6} + \mathbf{C_{10}H_{19}O_7} = C_{20}H_{38}O_{11} + O_2$$
$$\mathbf{C_{10}H_{19}O_6} + \mathbf{C_{10}H_{19}O_8} = C_{20}H_{38}O_{12} + O_2$$
$$\mathbf{C_{10}H_{19}O_7} + \mathbf{C_{10}H_{19}O_7} = C_{20}H_{38}O_{12} + O_2$$

As evident, the C20 ROOR accretion products are more complicated to interpret than the C13 ROOR, but the observation of these accretion products hints the formation of $C_{10}H_{19}O_3$, $C_{10}H_{19}O_5$, $C_{10}H_{19}O_6$, $C_{10}H_{19}O_7$ and $C_{10}H_{19}O_8$ radicals.

Supplementary Note 5: Atmospheric autoxidation initiated by TME+O_3

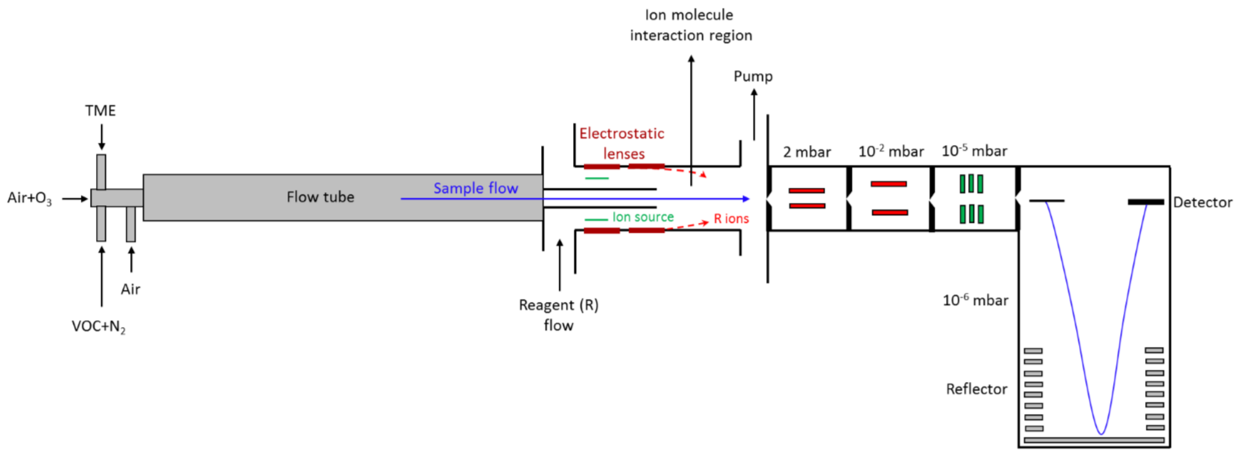

Supplementary Figure 7. Schematic of the Helsinki flow reactor system to study the VOC autoxidation at ~298 K. OH radicals were generated by the reaction of TME + O_3.

Beside decanal, the HOM monomers and ROOR accretion products were also observed during the autoxidation of methylcyclohexane, *n*-butylcyclohexane, cis-decalin, trans-decalin, heptanal., and 1-decanol. The corresponding mass spectra are shown in Supplementary Figure 8-12. In the case of the *n*-decane and 2,7-dimethyloctane, no HOM monomer and ROOR accretion products were measured (Supplementary Figure 13).

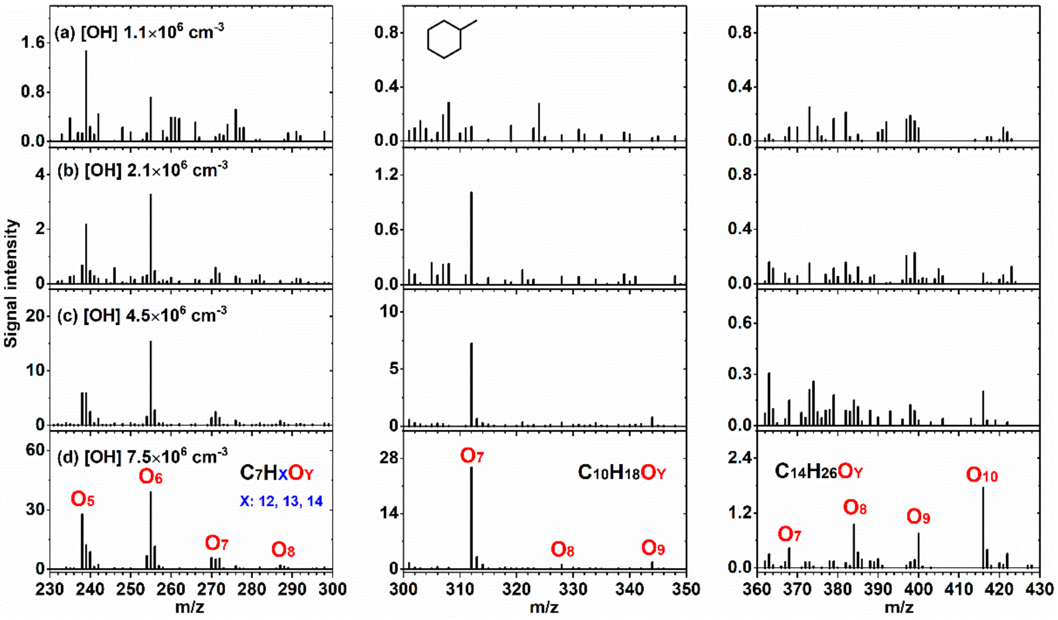

Supplementary Figure 8. Mass spectra of methylcyclohexane autoxidation in Helsinki flow reactor under different OH concentrations measured by the CI-APi-TOF. The left hand panels depict monomers and the middle and right hand panels the ROOR accretion products range for the same experiments. The TME + O_3 reaction is used to produce OH. The ROOR accretion products in the middle are produced from the bimolecular reactions of $C_3H_5O_3$ peroxy radical with the peroxy radicals from methylcyclohexane autoxidation. The ROOR accretion products on the right are produced from the bimolecular reactions of the peroxy radicals from methylcyclohexane autoxidation.

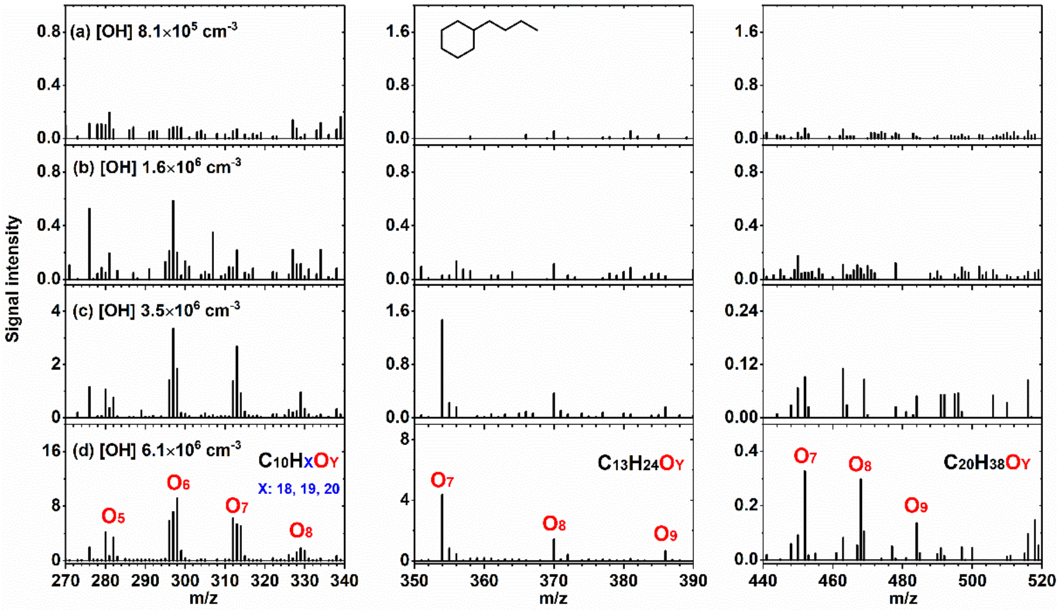

Supplementary Figure 9. Mass spectra of *n*-butylcyclohexane autoxidation in Helsinki flow reactor under different OH concentrations measured by the CI-APi-TOF. The left hand panels depict monomers and the middle and right hand panels the ROOR accretion products range for the same experiments. The TME + O_3 reaction is used to produce OH. The ROOR accretion products in the middle are produced from the bimolecular reactions of $C_3H_5O_3$ peroxy radical with the peroxy radicals from *n*-butylcyclohexane autoxidation. The ROOR accretion products on the right are produced from the bimolecular reactions of the peroxy radicals from *n*-butylcyclohexane autoxidation.

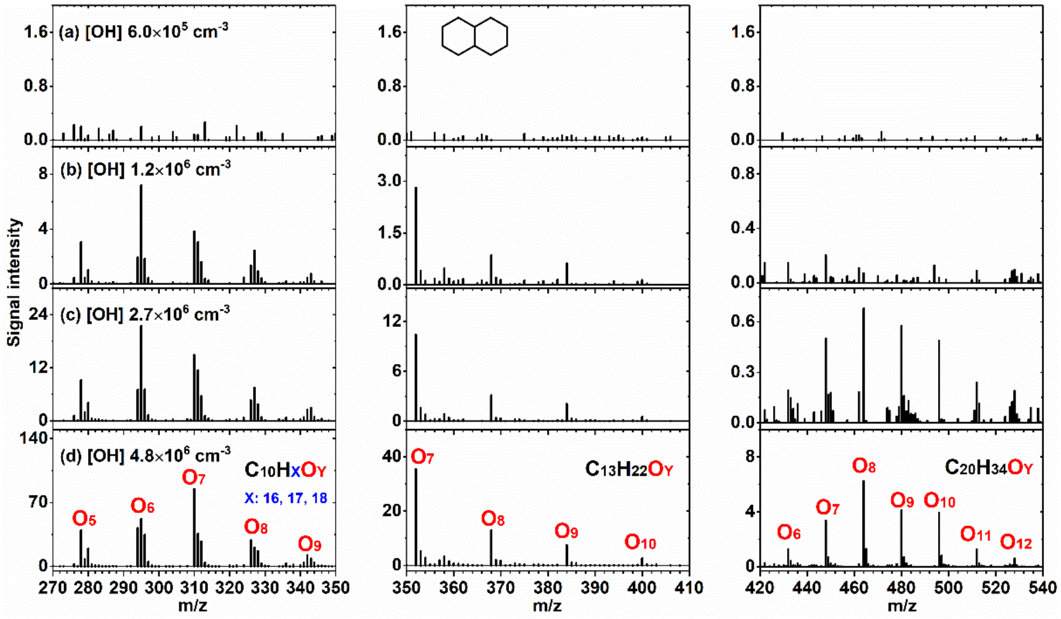

Supplementary Figure 10. Mass spectra of cis-decalin autoxidation in Helsinki flow reactor under different OH concentrations measured by the CI-APi-TOF. The left hand panels depict monomers and the middle and right hand panels the ROOR accretion products range for the same experiments. The TME + O_3 reaction is used to produce OH. The ROOR accretion products in the middle are produced from the bimolecular reactions of $C_3H_5O_3$ peroxy radical with the peroxy radicals from cis-decalin autoxidation. The ROOR accretion products on the right are produced from the bimolecular reactions of the peroxy radicals from cis-decalin autoxidation.

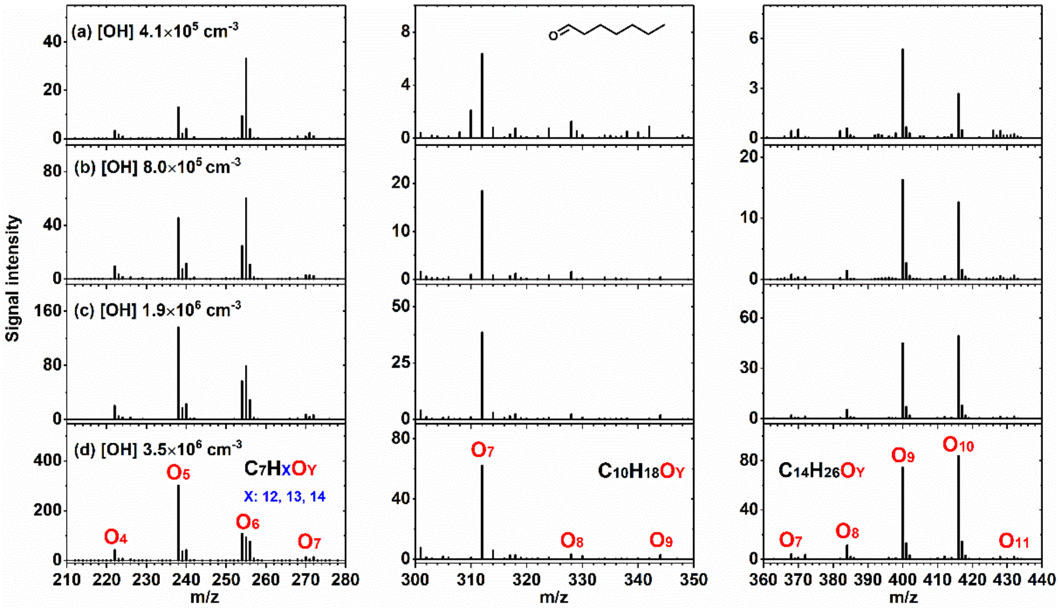

Supplementary Figure 11. Mass spectra of heptanal autoxidation in Helsinki flow reactor under different OH concentrations measured by the CI-APi-TOF. The left hand panels depict monomers and the middle and right hand panels the ROOR accretion products range for the same experiments. The TME + O_3 reaction is used to produce OH. The ROOR accretion products in the middle are produced from the bimolecular reactions of $C_3H_5O_3$ peroxy radical with the peroxy radicals from heptanal autoxidation. The ROOR accretion products on the right are produced from the bimolecular reactions of the peroxy radicals from heptanal autoxidation.

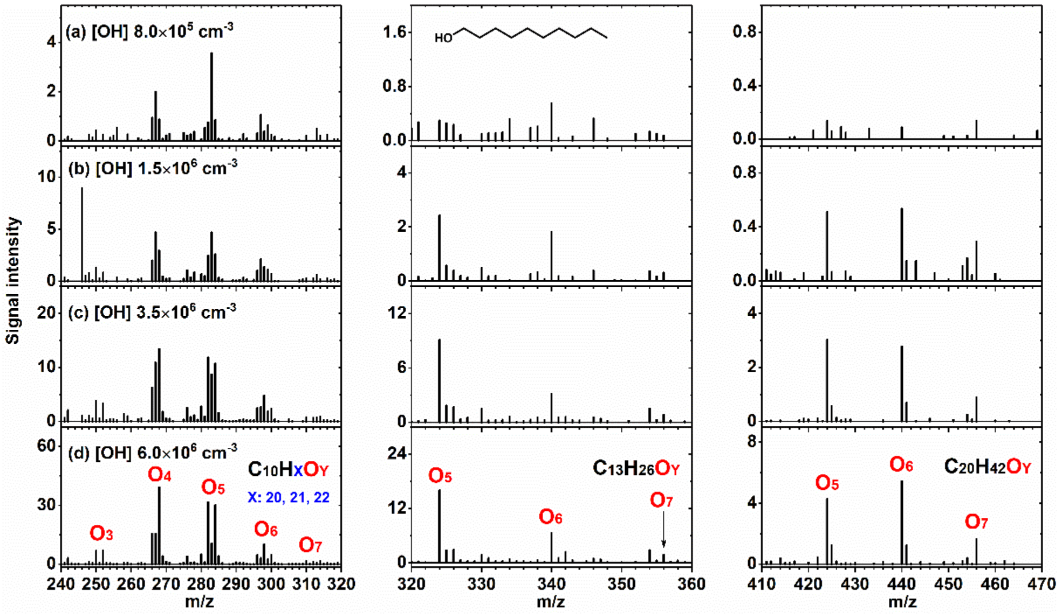

Supplementary Figure 12. Mass spectra of 1-decanol autoxidation in Helsinki flow reactor under different OH concentrations measured by the CI-APi-TOF. The left hand panels depict monomers and the middle and right hand panels the ROOR accretion products range for the same experiments. The TME + O_3 reaction is used to produce OH. The ROOR accretion products in the middle are produced from the bimolecular reactions of $C_3H_5O_3$ peroxy radical with the peroxy radicals from 1-decanol autoxidation. The ROOR accretion products on the right are produced from the bimolecular reactions of the peroxy radicals from 1-decanol autoxidation.

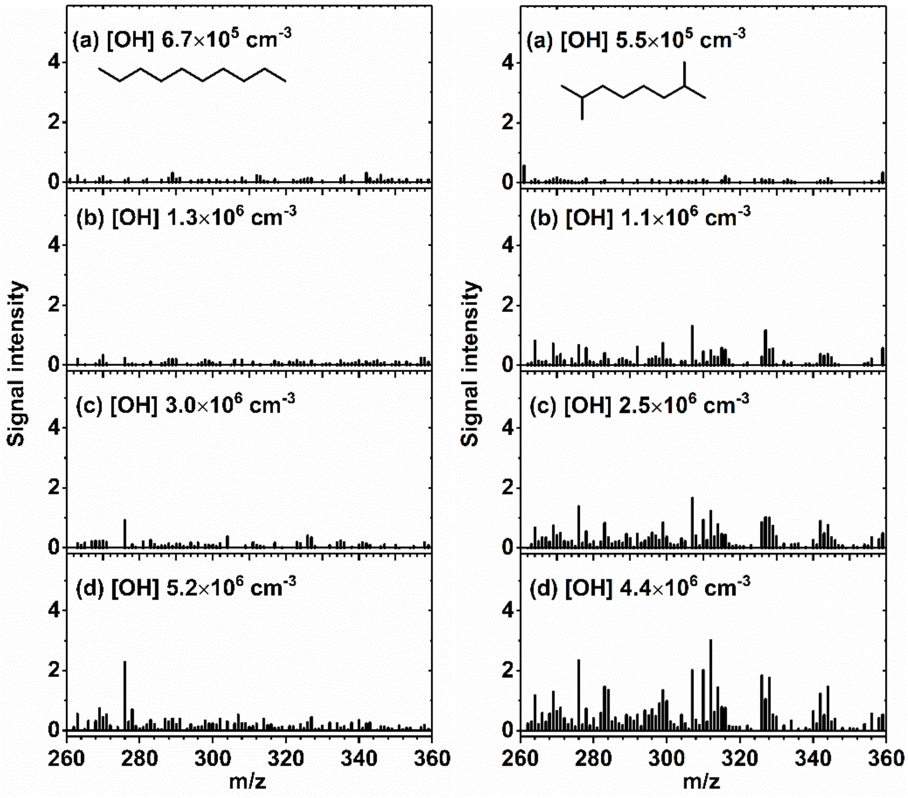

Supplementary Figure 13. Mass spectra of *n*-decane (left) and 2,7-dimethyloctane (right) autoxidation in Helsinki flow reactor under different OH concentrations measured by the CI-APi-TOF. The TME + O_3 reaction is used to produce OH.

Supplementary Note 6: Estimation of HOM yield in Helsinki flow reactor data

The quantification of the HOM yield used the measured [HOM] by CI-APi-TOF, measured O_3 concentration, and calculated starting mixing ratio of TME and VOC. Lacking good calibration standards for HOM, the CI-APi-TOF was only indirectly calibrated via the comparison to HOM measured in the α-pinene ozonolysis system. We assumed a 5% molar yield of HOM from this system, similar to the method of Riva et al. [19], based on earlier reports from this system [3,7]. The sensitivity of the CI-APi-TOF was then scaled to produce a 5 % HOM yield from this system. It is clear that this approach brings a large uncertainty range to the quantification of the results, estimated as at least -50%/+100%. However, this uncertainty in the absolute concentrations does not have a large impact on the conclusions of this work, as the relative uncertainty in the concentrations between different VOC is expected to be much lower, on the order of 10%. In order to obtain the HOM yields for the measurements with different VOC types and TME concentrations, we iteratively modelled the evolution of TME, O_3, OH, VOC and HOM concentration as a function of residence time in the flow reactor. The time step of the iteration was 0.0001 s and the model ran for 3 s, i.e. the residence time in the flow reactor before the sample reached CI-APi-TOF. We adjusted the HOM yield in the model until the modelled HOM concentration at t = 3 s matched the measured concentration. HOM concentration in time point t was calculated using the following equation:

$$HOM_t = HOM_{t-1} + step \times (\gamma_{HOM} k_{OH} VOC_{t-1} OH_{t-1} - k_{wall} HOM_{t-1})$$

where HOM_{t-1} is the HOM concentration in the previous time step, k_{OH} is the reaction rate coefficient at 298 K (see Supplementary Table 1), VOC_{t-1} and OH_{t-1} is the VOC and OH concentrations in the previous time step, respectively, and k_{wall} is the wall loss rate coefficient, estimated to be 0.2 s^{-1} for non-volatile vapors in our flow reactor. The OH concentration in the model was calculated from the TME+O_3 reaction using k_{O3+TME}= 1.0×10^{-15} and k_{OH+TME} = 1.0×10^{-10} cm^3/molecule/s. The equations for other reactions are:

$$VOC_t = VOC_{t-1} + step \times (-VOC_{t-1} k_{OH+VOC} OH_{t-1})$$

$$TME_t = TME_{t-1} + step \times (-TME_{t-1} k_{O3+TME} O3_{t-1} - TME_{t-1} k_{OH+TME} OH_{t-1})$$

$$O3_t = O3_{t-1} + step \times (-TME_{t-1} k_{O3+TME} O3_{t-1})$$

$$OH_t = OH_{t-1} + step \times (TME_{t-1} k_{O3+TME} O3_{t-1} - TME_{t-1} k_{OH+TME} OH_{t-1} - VOC_{t-1} k_{OH+VOC} OH_{t-1})$$

Supplementary Table 1. Rate constant of selected VOC with OH at 298 K.

Name	Formula	structure	k(cm^3/molecule/s)	Reference
methylcyclohexane	C_7H_{14}	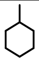	1.1×10^{-11}	[20]
n-butylcyclohexane	$C_{10}H_{20}$	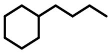	1.47×10^{-11}	[21]
cis-decalin	$C_{10}H_{18}$	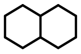	2.01×10^{-11}	[22]
trans-decalin	$C_{10}H_{18}$	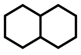	2.06×10^{-11}	[22]
heptanal	$C_7H_{14}O$	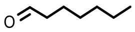	3.0×10^{-11}	[23]
decanal	$C_{10}H_{20}O$	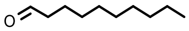	3.0×10^{-11}	est from heptanal
1-decanol	$C_{10}H_{22}O$	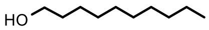	1.5×10^{-11}	est

Supplementary Note 7: Kinetic modelling of decanal and decalin oxidation in flow reactors

The short residence times (3 s in Helsinki, 7.9 s in Leipzig) and fairly low amounts of reacted VOC in the flow reactors will impact the observed products. As the oxidation rates increase, the concentration of RO_2 radicals increase, which leads to an increase in RO_2 cross reactions in a non-linear fashion. For example, products requiring several bimolecular reactions are expected to increase more rapidly at higher oxidation rates compared to products requiring only one, or none at all. For interpreting different patterns and slopes in the data, even very simple kinetic models can provide useful information. In this work, two separate models were used: a "lumped" model for modeling decanal and decalin in the Helsinki flow reactor, and an "explicit" model for decalin in the Leipzig experiments. Details of each model and the results are discussed below.

"Lumped" model for Helsinki experiments

The combination of short residence time and low [OH] (in the range 10^5 – 10^7 molecules cm^{-3}) in the Helsinki flow reactor meant that multi-generation OH oxidation was negligible. Therefore, the explanation for the steeply rising yields of cycloalkanes as a function of reacted VOC needed to be sought elsewhere. To explore this, we built a simple model for constraining the reaction kinetics able to produce the general trends observed in Fig. 3. We included only the most basic VOC-OH and RO_2 reaction pathways.

We allowed the initial ("primary") peroxy radical, $R'O_2$, to form R′O via bimolecular reaction with other RO_2. The R′O could then further isomerize to a secondary $R''O_2$. Upon reaction with RO_2, the $R''O_2$ could form R″O, which would isomerize to a tertiary $R'''O_2$. The reaction schemes for all included compounds formed in the flow reactor are listed below. The reactions of compounds without a source in the reactor, namely TME, VOC, and O_3, are excluded from the list, though their loss terms are found in the reactions below as source terms for other products. $R^{Tot}O_2$ is the sum of all RO_2 radicals in the flow reactor.

$$\frac{dOH}{dt} = k_{O_3+TME} \cdot TME \cdot O_3 - k_{OH+TME} \cdot TME \cdot OH - k_{OH+VOC} \cdot VOC \cdot OH$$
$$\frac{dR^{TME}O_2}{dt} = k_{O_3+TME} \cdot TME \cdot O_3 - k_{RO_2+RO_2} \cdot R^{TME}O_2 \cdot R^{Tot}O_2 - k_{wall}R^{TME}O_2$$
$$\frac{dR'O_2}{dt} = k_{OH+VOC} \cdot VOC \cdot OH - k_{RO_2+RO_2} \cdot R'O_2 \cdot R^{Tot}O_2 - k_{wall}R'O_2$$
$$\frac{dR''O_2}{dt} = \gamma_{sec} \cdot k_{RO_2+RO_2} \cdot R'O_2 \cdot R^{Tot}O_2 - k_{RO_2+RO_2} \cdot R''O_2 \cdot R^{Tot}O_2 - k_{wall}R''O_2$$
$$\frac{dR'''O_2}{dt} = \gamma_{tri} \cdot k_{RO_2+RO_2} \cdot R''O_2 \cdot R^{Tot}O_2 - k_{RO_2+RO_2} \cdot R'''O_2 \cdot R^{Tot}O_2 - k_{wall}R'''O_2$$
$$\frac{dProd1}{dt} = (1-\gamma_{sec}) \cdot k_{RO_2+RO_2} \cdot R'O_2 \cdot R^{Tot}O_2 - k_{wall} \cdot Prod1$$
$$\frac{dProd2}{dt} = (1-\gamma_{tri}) \cdot k_{RO_2+RO_2} \cdot R''O_2 \cdot R^{Tot}O_2 - k_{wall} \cdot Prod2$$
$$\frac{dProd3}{dt} = k_{RO_2+RO_2} \cdot R'''O_2 \cdot R^{Tot}O_2 - k_{wall} \cdot Prod3$$

In the reactions, focus was on the kinetics of RO_2+RO_2 reactions. No autoxidation was included in the model, and this implicitly means that e.g. $R'O_2$ signifies a "lumped" group of many radicals with different levels of oxidation. The fractions of each radical group ($R'O_2$, $R''O_2$ and $R'''O_2$) that have undergone autoxidation to form HOM products

(Prod1, Prod2 and Prod3) are free parameters in the model (see below). The common denominator for all radicals in a specific group is they have undergone the same amount of RO isomerization steps (0 for $R'O_2$, 1 for $R''O_2$, and 2 for $R'''O_2$). An overview of the main reactions is given in Supplementary Figure 14a.

The free parameters in the model were thus

- The yields (y_sec and y_tri) giving the branching ratios of reformed RO_2 after RO isomerization following an RO_2+RO_2 reaction. In our runs, these values were kept at 60% as a typical RO yield from RO_2+RO_2 reactions.
- The reaction rate coefficient $k_{RO2+RO2}$. In our runs it was kept at 2×10^{-11} cm^3 s^{-1} in all cases except when the primary, non-functionalized $R'O_2$ from decalin + OH was a reaction partner. In those cases the rate was ten times slower. These rates are loosely based on rates given by Berndt et al. [9].
- The fraction of each group of closed shell products (Prod1-3) that counted as HOM. This signifies the fraction that underwent sufficient autoxidation to obtain a high enough oxygen content. These values are not included in the reactions above, since they were only scaling parameters applied after the model had been run.
- The fraction of different generation RO_2 that are detectable.

With this very simple model, and with reasonable values for the selected free parameters, we were able to match the trends observed in Fig. 3 for the test cases of decanal and decalin (Supplementary Figure 14b & 14c). We also emphasize that the simplicity of the model also means that it is relatively well constrained, and thus cannot be tuned to match any arbitrary trends. Decanal showed a fairly constant yield as a function of reacted VOC (Fig. 3), and this is captured by the model if we assume that 15% of $R''O_2$ and 15% of Products2 (see Supplementary Figure 14a) are detected as HOM. In other words, all the observed HOM in our model have undergone one RO isomerization step. In addition to matching the observed yields, the model result also matches the data in the sense that at the lower reaction rates, the signal of $R''O_2$ is the largest contributor in the model, as is the case in the experiments (see Supplementary Figure 5), while at higher reaction rates the closed shell products become more important.

In the case of decalin, the strongly increasing trend in Fig. 3 could also be matched (Supplementary Figure 14c), though with quite different parameters. The model result assumed that 13% of the $R'''O_2$ and 13% of the Products3 (see Supplementary Figure 14a) were detected as HOM. Thus, HOM formation from decalin can be explained by requiring two RO isomerization steps in order to produce HOM, thereby producing a much steeper trend than e.g. decanal in Fig. 3.

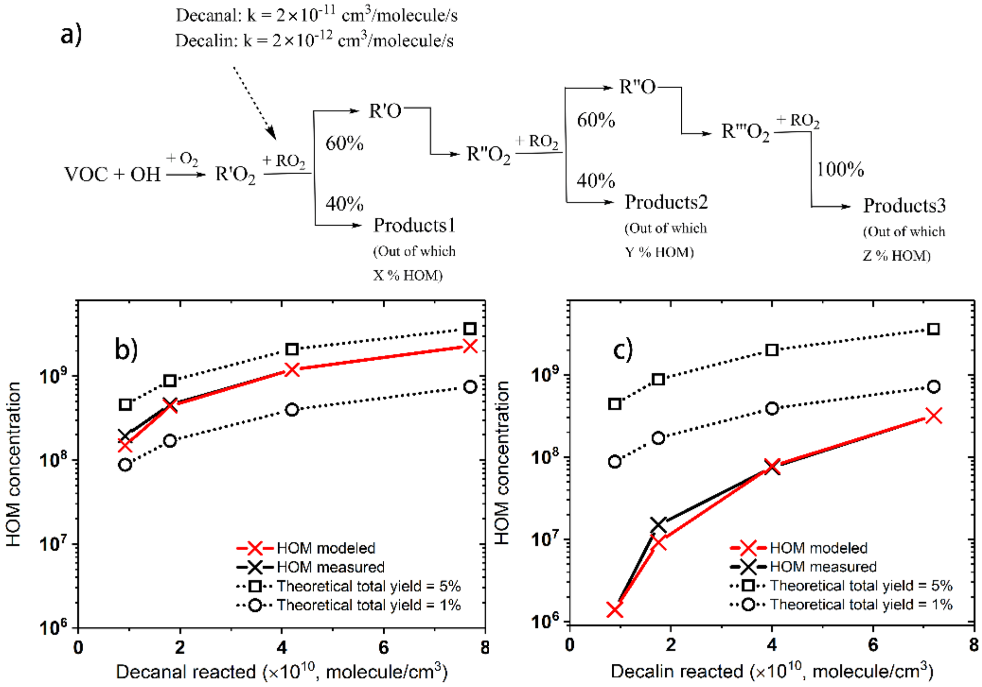

Supplementary Figure 14. a) Main reaction pathways used in the "lumped" kinetic model used for the Helsinki flow reactor. b & c) Modeled vs measured HOM concentrations during decanal (b) and decalin (c) oxidation in the Helsinki flow reactor. The dashed lines show the 1% and 5 % HOM yields. See text for details on parameters used in the model. Note, this plot shows HOM concentration rather than the HOM yields in Fig. 3.

"Explicit" model for Leipzig experiments

Here we attempted to model the Leipzig experiments where the autoxidation of decalin was initiated by OH from TME+O_3. Ozone was produced by passing 2 L min^{-1} (STP) air through two ozone generators (UVP OG-2) working in parallel and blended with additional air to a total flow of 5 L min^{-1} (STP) taken as the feed for the inner tube. All gas flows were set by means of calibrated gas flow controllers (MKS 1259/1179). Ozone concentrations were measured at the outflow of the reactor by a gas monitor (Thermo Environmental Instruments 49C). A proton transfer reaction - mass spectrometer (PTR-MS; HS PTR-QMS 500, Ionicon) served as an on-line monitor for organic compounds.

The initial decalin concentration was 5×10^{12} molecules/cm^3 and TME 1×10^{11} molecules/cm^3. The O_3 was varied in the range of (6.7-200)×10^{10} molecules/cm^3 to get different OH radical concentrations. The residence time of the reaction is 7.9 s. The rate constant of k_{O3+TME} is 1×10^{-15} cm^3/molecule/s, $k_{OH+decalin}$ is 2×10^{-11} cm^3/molecule/s, and the wall loss rate was assumed negligible (k_{wall} is 0 s^{-1}).

The observed peroxy radicals, as a function of reacted decalin, are shown as markers in Supplementary Figure 15. $C_{10}H_{17}O_2$ is the primarily formed RO_2 radical from decalin + OH, and showed a linear increase with rising decalin

conversion. The signals of the $C_{10}H_{17}O_3$, $C_{10}H_{17}O_5$ and $C_{10}H_{17}O_7$ radicals show slopes of about 2, indicating the reaction of $C_{10}H_{17}O_2$ with any RO_2 as the rate-limiting step for their production. $C_{10}H_{17}O_3$ is most likely an aldehyde-containing secondary peroxy radical formed after ring-opening (C-C scission) of the RO radical arising from the $C_{10}H_{17}O_2$ reaction with RO_2. $C_{10}H_{17}O_5$ and $C_{10}H_{17}O_7$ can be formed following one and two H-shifts and subsequent O_2 additions, respectively. Finally, $C_{10}H_{17}O_6$, $C_{10}H_{17}O_8$, and $C_{10}H_{17}O_9$ are also formed at low concentrations, showing steeper slopes than the other peroxy radicals, pointing to one or more RO isomerization steps.

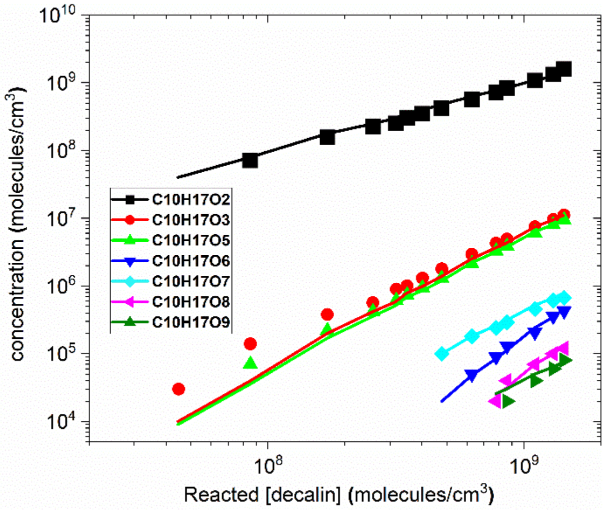

Supplementary Figure 15. Experiment measurement (symbols) and model simulation (lines) of peroxy radicals in decalin autoxidation. The OH radical is formed through the TME + O_3 reaction. The measured concentration of $C_{10}H_{17}O_2$ was multiplied by 600, assuming its concentration is underestimated. This value is estimated from the reacted decalin, since under the experiment condition, all the decalin is converted into $C_{10}H_{17}O_2$.

Based on these observations, we built a kinetic model for decalin, where each RO_2 and its reactions are included separately. The reactions in the model are shown below. For each peroxy radical, the bimolecular reactions are dominated by the "primary" RO_2 from the decalin + OH reaction and the $CH_3C(O)CH_2O_2$ radical from TME + O_3. Thus, only the bimolecular reactions with these two radicals, and itself were included in the model. The branching ratio of RO_2+RO_2 to form ROs was assumed to be 0.6. In the scheme, the notation RO_X refers to the peroxy radical $C_{10}H_{17}O_X$. RO_3 and RO_4 are formed via alkoxy steps, while the more oxygenated RO_X in the model are formed by (multistep) peroxy radical H-shifts from RO_3 and RO_4.

$$\frac{dOH}{dt} = k_{O_3+TME} \cdot TME \cdot O_3 - k_{OH+TME} \cdot TME \cdot OH - k_{OH+VOC} \cdot VOC \cdot OH$$

$$\frac{dTME}{dt} = -k_{O_3+TME} \cdot TME \cdot O_3 - k_{OH+TME} \cdot TME \cdot OH$$

$$\frac{dC3H5O3}{dt} = k_{O_3+TME} \cdot TME \cdot O_3 - 2 \cdot k_{C3H5O3+C3H5O3} \cdot C3H5O3 - k_{wall} \cdot C3H5O3$$

$$\frac{dVOC}{dt} = -k_{OH+VOC} \cdot VOC \cdot OH$$

$$\frac{dRO2}{dt} = k_{OH+VOC} \cdot VOC \cdot OH - 2 \cdot k_{RO2+RO2} \cdot RO2 \cdot RO2 - k_{RO2+C3H5O3} \cdot RO2 \cdot C3H5O3 - k_{wall} \cdot RO2$$

$$\frac{dRO3}{dt} = 0.6 \cdot 2 \cdot k_{RO2+RO2} \cdot RO2 \cdot RO2 + 0.6 \cdot k_{RO2+C3H5O3} \cdot RO2 \cdot C3H5O3 - 2 \cdot k_{RO3+RO3} \cdot RO3 \cdot RO3 - k_{RO2+RO3} \cdot RO2 \cdot RO3 - k_{RO3+C3H5O3} \cdot RO3 \cdot C3H5O3 - k_{wall} \cdot RO3$$

$$\frac{dRO4}{dt} = 0.6 \cdot 2 \cdot k_{RO3+RO3} \cdot RO3 \cdot RO3 + 0.6 \cdot k_{RO2+RO3} \cdot RO2 \cdot RO3 + 0.6 \cdot k_{RO2+C3H5O3} \cdot RO2 \cdot C3H5O3 - 2 \cdot k_{RO4+RO4} \cdot RO4 \cdot RO4 - k_{RO2+RO4} \cdot RO2 \cdot RO4 - k_{RO4+RC3H5O3} \cdot RO4 \cdot C3H5O3 - k_{wall} \cdot RO4$$

$$\frac{dRO5}{dt} = k_{RO3} \cdot RO3 - 2 \cdot k_{RO5+RO5} \cdot RO5 \cdot RO5 - k_{RO2+RO5} \cdot RO2 \cdot RO5 - k_{RO5+C3H5O3} \cdot RO5 \cdot C3H5O3 - k_{wall} \cdot RO5$$

$$\frac{dRO6}{dt} = k_{RO4} \cdot RO4 - 2 \cdot k_{RO6+RO6} \cdot RO6 \cdot RO6 - k_{RO2+RO6} \cdot RO2 \cdot RO6 - k_{RO6+C3H5O3} \cdot RO6 \cdot C3H5O3 - k_{wall} \cdot RO6$$

$$\frac{dRO7}{dt} = k_{RO5} \cdot RO5 - 2 \cdot k_{RO7+RO7} \cdot RO7 \cdot RO7 - k_{RO2+RO7} \cdot RO2 \cdot RO7 - k_{RO7+C3H5O3} \cdot RO7 \cdot C3H5O3 - k_{wall} \cdot RO7$$

$$\frac{dRO8}{dt} = k_{RO6} \cdot RO6 - 2 \cdot k_{RO8+RO8} \cdot RO8 \cdot RO8 - k_{RO2+RO8} \cdot RO2 \cdot RO8 - k_{RO8+C3H5O3} \cdot RO8 \cdot C3H5O3 - k_{wall} \cdot RO8$$

$$\frac{dRO9}{dt} = k_{RO7} \cdot RO7 - 2 \cdot k_{RO9+RO9} \cdot RO9 \cdot RO9 - k_{RO2+RO9} \cdot RO2 \cdot RO9 - k_{RO9+C3H5O3} \cdot RO9 \cdot C3H5O3 - k_{wall} \cdot RO9$$

The rate constant for the related reactions were estimated, and given below.

(1) $k_{RO3} = 0.4\ s^{-1}$; $k_{RO4} = 0.5\ s^{-1}$; $k_{RO5} = 0.05\ s^{-1}$; $k_{RO6} = 0.25\ s^{-1}$; $k_{RO7} = 0.04\ s^{-1}$.

(2) $k_{RO2+RO2} = 2.5\times10^{-12}\ cm^3$/molecule/s; $k_{RO3+RO3} = 2\times10^{-11}\ cm^3$/molecule/s; $k_{RO4+RO4} = 2\times10^{-11}\ cm^3$/molecule/s; $k_{RO5+RO5} = 2\times10^{-11}\ cm^3$/molecule/s; $k_{RO6+RO6} = 2\times10^{-11}\ cm^3$/molecule/s; $k_{RO7+RO7} = 2\times10^{-11}\ cm^3$/molecule/s; $k_{RO8+RO8} = 2\times10^{-11}\ cm^3$/molecule/s.

(3) $k_{RO2+RO3} = 2\times10^{-11}\ cm^3$/molecule/s; $k_{RO2+RO4} = 2\times10^{-11}\ cm^3$/molecule/s; $k_{RO2+RO5} = 2\times10^{-11}\ cm^3$/molecule/s; $k_{RO2+RO6} = 2\times10^{-11}\ cm^3$/molecule/s; $k_{RO2+RO7} = 2\times10^{-11}\ cm^3$/molecule/s; $k_{RO2+RO8} = 2\times10^{-11}\ cm^3$/molecule/s; $k_{RO2+RO9} = 2\times10^{-11}\ cm^3$/molecule/s.

(4) $k_{C3H5O3+C3H5O3} = 2.5\times10^{-12}\ cm^3$/molecule/s; $k_{C3H5O3+RO2} = 2.5\times10^{-12}\ cm^3$/molecule/s; $k_{C3H5O3+RO3} = 2\times10^{-11}\ cm^3$/molecule/s; $k_{C3H5O3+RO4} = 2\times10^{-11}\ cm^3$/molecule/s; $k_{C3H5O3+RO5} = 2\times10^{-11}\ cm^3$/molecule/s; $k_{C3H5O3+RO6} = 2\times10^{-11}\ cm^3$/molecule/s; $k_{C3H5O3+RO7} = 2\times10^{-11}\ cm^3$/molecule/s; $k_{C3H5O3+RO8} = 2\times10^{-11}\ cm^3$/molecule/s; $k_{C3H5O3+RO9} = 2\times10^{-11}\ cm^3$/molecule/s.

Supplementary Note 8: Autoxidation of decalin, cyclohexane, and n-decane in the presence of NO conducted in the Leipzig flow reactor

In the Leipzig flow reactor, the autoxidation of decalin, cyclohexane and *n*-decane was studied. OH radicals were formed through the photolysis of isopropyl nitrite with an initial nitrite concentration of 2.7×10^{11} molecules/cm^3. In this experiment, the flow system was surrounded by 8 NARVA 36W Blacklight Blue lamps, emitting in the range 350 - 400 nm for homogeneous illumination of the whole reaction zone. Synthesis and purification of isopropyl nitrite was carried out as described elsewhere [24]. Isopropyl nitrite photolysis generates i-C_3H_7O and NO with subsequent reaction of i-C_3H_7O with O_2 forming HO_2 radicals and acetone. Finally, NO reacts with HO_2 forming OH radicals and NO_2.

Compared to the conditions of the TME + O_3 reaction, the photolysis experiments with isopropyl nitrite generated higher OH radical levels. NO was added to the reaction system with concentrations up to 2.4×10^{11} molecules/cm^3. The sharp increase of all products at the lowest added NO steps is primarily due to an increased OH concentration, following an enhanced NO + HO_2 $\rightarrow$ NO_2 + OH reaction. The lower limit concentration of RO_2 radicals, carbonyls, and organic nitrates as a function of NO are shown in Supplementary Figure 16-18. The corresponding mass spectra at the highest NO concentration of 2.4×10^{11} molecules/cm^3 are presented in Fig. 4.

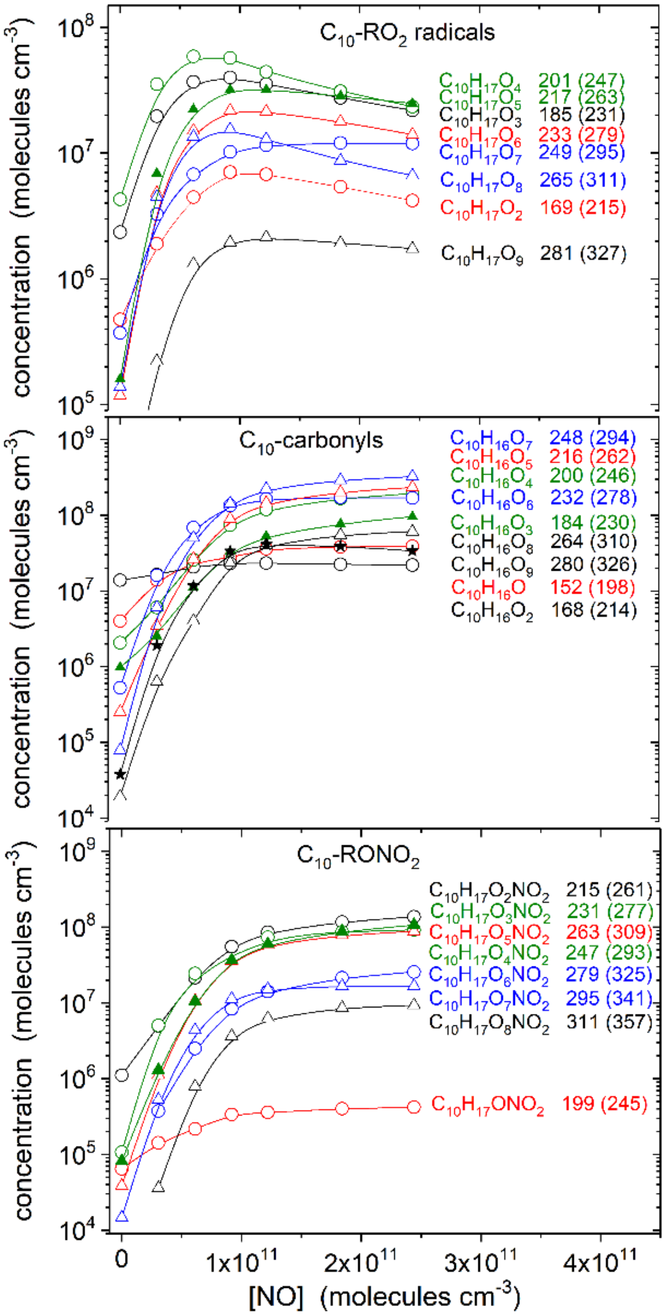

Supplementary Figure 16. Lower limit concentration of RO_2 radicals, carbonyls, and organic nitrates as a function of NO observed from decalin autoxidation. The residence time is 7.9 s, temperature of the reactor is 293 K, and the relative humidity is <0.1%. OH radicals have been formed through the photolysis of isopropyl nitrite with an initial nitrite concentration of 2.7×10^{11} molecules/cm^3. The initial concentration of decalin is 2×10^{12} molecules/cm^3. The integer mass of each molecule is written next to its chemical formula. The number in brackets is the mass of the molecule with the reagent ion ($C_2H_5NH_3^+$, m/z 46).

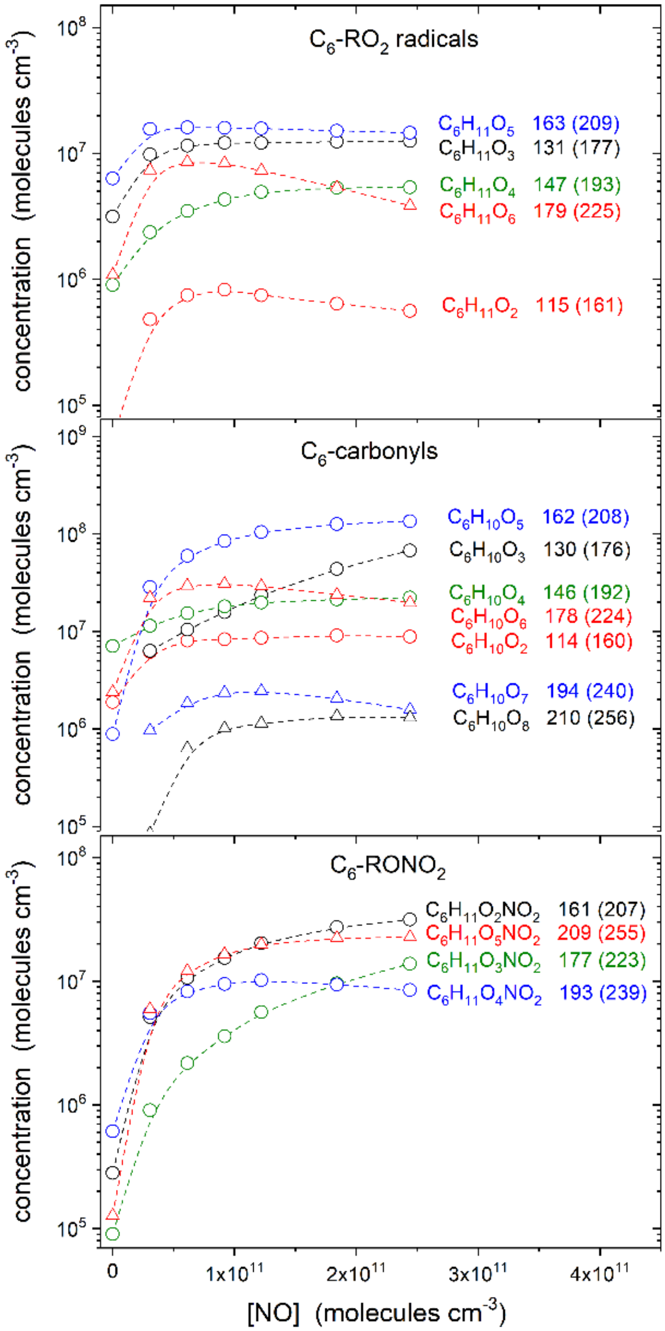

Supplementary Figure 17. Lower limit concentration of RO_2 radicals, carbonyls, and organic nitrates as a function of NO observed from cyclohexane autoxidation. The residence time is 7.9 s, temperature of the reactor is 293 K, and the relative humidity is < 0.1%. OH radicals have been formed through the photolysis of isopropyl nitrite with an initial nitrite concentration of 2.7×10^{11} molecules/cm^3. The initial concentration of cyclohexane is 1.5×10^{13} molecules/cm^3. The integer mass of each molecule is written next to its chemical formula. The number in brackets is the mass of the molecule with the reagent ion ($C_2H_5NH_3^+$, m/z 46).

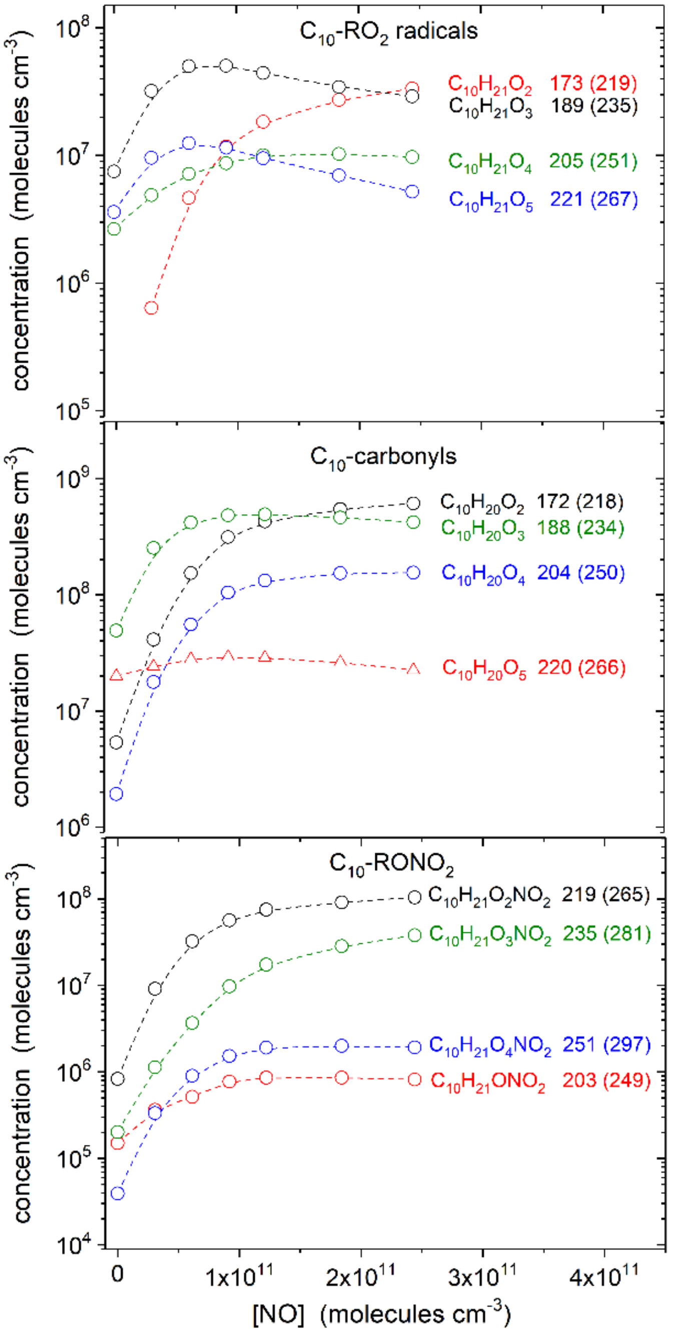

Supplementary Figure 18. Lower limit concentration of RO_2 radicals, carbonyls, and organic nitrates as a function of NO observed from *n*-decane autoxidation. The residence time is 7.9 s, temperature of the reactor is 298 K, and the relative humidity is < 0.1%. OH radicals have been formed through the photolysis of isopropyl nitrite with an initial nitrite concentration of 2.7×10^{11} molecules/cm^3. The initial concentration of *n*-decane is 3.5×10^{12} molecules/cm^3. The integer mass of each molecule is written next to its chemical formula. The number in brackets is the mass of the molecule with the reagent ion ($C_2H_5NH_3^+$, m/z 46).

Supplementary References

1 Crounse, J. D., Nielsen, L. B., Jørgensen, S., Kjaergaard, H. G. & Wennberg, P. O. Autoxidation of Organic Compounds in the Atmosphere. *J. Phys. Chem. Lett.* **4**, 3513-3520, doi:10.1021/jz4019207 (2013).

2 Jenkin, M. E., Valorso, R., Aumont, B. & Rickard, A. R. Estimation of rate coefficients and branching ratios for reactions of organic peroxy radicals for use in automated mechanism construction. *Atmos. Chem. Phys.* **19**, 7691-7717, doi:10.5194/acp-19-7691-2019 (2019).

3 Ehn, M. *et al.* A large source of low-volatility secondary organic aerosol. *Nature* **506**, 476-479, doi:10.1038/nature13032 (2014).

4 Jokinen, T. *et al.* Production of extremely low volatile organic compounds from biogenic emissions: Measured yields and atmospheric implications. *Proc. Natl. Acad. Sci. U.S.A.* **112**, 7123-7128, doi:10.1073/pnas.1423977112 (2015).

5 Molteni, U. *et al.* Formation of highly oxygenated organic molecules from aromatic compounds. *Atmos. Chem. Phys.* **18**, 1909-1921, doi:10.5194/acp-18-1909-2018 (2018).

6 Wang, Z. *et al.* Unraveling the structure and chemical mechanisms of highly oxygenated intermediates in oxidation of organic compounds. *Proc. Natl. Acad. Sci. U.S.A.* **114**, 13102–13107, doi:10.1073/pnas.1707564114 (2017).

7 Jokinen, T. *et al.* Rapid Autoxidation Forms Highly Oxidized RO_2 Radicals in the Atmosphere. *Angew. Chem. Int. Ed.* **53**, 14596-14600, doi:10.1002/anie.201408566 (2014).

8 Master Chemical Mechanism, MCM v3.3.1, http://mcm.leeds.ac.uk/MCM.

9 Berndt, T. *et al.* Accretion Product Formation from Self- and Cross-Reactions of RO_2 Radicals in the Atmosphere. *Angew. Chem. Int. Ed.* **57**, 3820-3824, doi:10.1002/anie.201710989 (2018).

10 Bianchi, F. *et al.* Highly Oxygenated Organic Molecules (HOM) from Gas-Phase Autoxidation Involving Peroxy Radicals: A Key Contributor to Atmospheric Aerosol. *Chem. Rev.* **119**, 3472-3509, doi:10.1021/acs.chemrev.8b00395 (2019).

11 Davis, A. C. & Francisco, J. S. Reactivity Trends within Alkoxy Radical Reactions Responsible for Chain Branching. *J. Am. Chem. Soc.* **133**, 18208-18219, doi:10.1021/ja204806b (2011).

12 Vereecken, L. & Peeters, J. Decomposition of substituted alkoxy radicals—part I: a generalized structure–activity relationship for reaction barrier heights. *Phys. Chem. Chem. Phys.* **11**, 9062-9074, doi:10.1039/B909712K (2009).

13 Vereecken, L. & Peeters, J. A structure–activity relationship for the rate coefficient of H-migration in substituted alkoxy radicals. *Phys. Chem. Chem. Phys.* **12**, 12608-12620, doi:10.1039/C0CP00387E (2010).

14 Calvert, J. G., Derwent, R. G., Orlando, J. J., Tyndall, G. S. & Wallington, T. J. *Mechanisms of atmospheric oxidation of the alkanes*. (Oxford University Press, 2008).

15 Zhou, Y. Z., Li, S., Li, Q. S. & Zhang, S. W. Theoretical investigation of the decarboxylation reaction of CH_3CO_2 radical. *J. Mol. Struct: THEOCHEM* **854**, 40-45, doi:https://doi.org/10.1016/j.theochem.2007.12.033 (2008).

16 Zádor, J., Taatjes, C. A. & Fernandes, R. X. Kinetics of elementary reactions in low-temperature autoignition chemistry. *Prog. Energy Combust. Sci.* **37**, 371-421, doi:http://dx.doi.org/10.1016/j.pecs.2010.06.006 (2011).

17 Wang, Z., Herbinet, O., Hansen, N. & Battin-Leclerc, F. Exploring hydroperoxides in combustion: History, recent advances and perspectives. *Prog. Energy Combust. Sci.* **73**, 132-181, doi:https://doi.org/10.1016/j.pecs.2019.02.003 (2019).

18 Rissanen, M. P. *et al.* The Formation of Highly Oxidized Multifunctional Products in the Ozonolysis of Cyclohexene. *J. Am. Chem. Soc.* **136**, 15596-15606, doi:10.1021/ja507146s (2014).

19 Riva, M. *et al.* Chemical transformations in monoterpene-derived organic aerosol enhanced by inorganic composition. *NPJ. Clim. Atmos. Sci.* **2**, 2, doi:10.1038/s41612-018-0058-0 (2019).

20 Baulch, D. L., Bowers, M., Malcolm, D. G. & Tuckerman, R. T. Evaluated Kinetic Data for High‐Temperature Reactions. Volume 5. Part 1. Homogeneous Gas Phase Reactions of the Hydroxyl Radical with Alkanes. *J. Phys. Chem. Ref. Data* **15**, 465-592, doi:10.1063/1.555774 (1986).

21 Atkinson, R. & Arey, J. Atmospheric Degradation of Volatile Organic Compounds. *Chem. Rev.* **103**, 4605-4638, doi:10.1021/cr0206420 (2003).

22 Atkinson, R., Aschmann, S. M. & Carter, W. P. L. Rate constants for the gas-phase reactions of OH radicals with a series of bi- and tricycloalkanes at 299 ± 2 K: Effects of ring strain. *Int. J. Chem. Kinet.* **15**, 37-50,

doi:10.1002/kin.550150105 (1983).
23 Albaladejo, J., Ballesteros, B., Jiménez, E., Martín, P. & Martínez, E. A PLP–LIF kinetic study of the atmospheric reactivity of a series of C4–C7 saturated and unsaturated aliphatic aldehydes with OH. *Atmos. Environ.* **36**, 3231-3239, doi:https://doi.org/10.1016/S1352-2310(02)00323-0 (2002).
24 Raff, J. D. & Finlayson-Pitts, B. J. Hydroxyl Radical Quantum Yields from Isopropyl Nitrite Photolysis in Air. *Environ. Sci. Technol.* **44**, 8150-8155, doi:10.1021/es102218d (2010).
